# Unravelling Novel Roles of Salivary Exosomes in the Regulation of Human Corneal Stromal Cell Migration and Wound Healing

**DOI:** 10.3390/ijms23084330

**Published:** 2022-04-14

**Authors:** Paulina Escandon, Angela Liu, Sarah E. Nicholas, Asher Khan, Kamran M. Riaz, Dimitrios Karamichos

**Affiliations:** 1 North Texas Eye Research Institute, University of North Texas Health Science Center, 3430 Camp Bowie Blvd, Fort Worth, TX 76107, USA; paulina.escandon@unthsc.edu (P.E.); angelaliu@my.unthsc.edu (A.L.); sarah.nicholas@unthsc.edu (S.E.N.); 2 Department of Pharmaceutical Sciences, University of North Texas Health Science Center, 3500 Camp Bowie Blvd, Fort Worth, TX 76107, USA; 3 Texas College of Osteopathic Medicine, University of North Texas Health Science Center, 3500 Camp Bowie Blvd, Fort Worth, TX 76107, USA; 4 Department of Ophthalmology, Dean McGee Eye Institute, University of Oklahoma Health Sciences Center, Oklahoma City, OK 73104, USA; asher-khan@ouhsc.edu (A.K.); kamran-riaz@dmei.org (K.M.R.); 5 Department of Pharmacology and Neuroscience, University of North Texas Health Science Center, 3500 Camp Bowie Blvd, Fort Worth, TX 76107, USA

**Keywords:** salivary exosomes, ocular diseases, corneal wound healing, cell migration, fibrosis, diabetes mellitus, keratoconus

## Abstract

Salivary exosomes have demonstrated vast therapeutic and diagnostic potential in numerous diseases. This study pioneers previously unexplored roles of SE in the context of corneal wound healing by utilizing primary corneal stromal cells from healthy (HCFs), type I diabetes mellitus (T1DMs), type II DM (T2DMs), and keratoconus (HKCs) subjects. Purified, healthy human SEs carrying tetraspanins CD9+, CD63+, and CD81+ were utilized. Scratch and cell migration assays were performed after 0, 6, 12, 24, and 48 h following SE stimulation (5 and 25 µg/mL). Significantly slower wound closure was observed at 6 and 12 h in HCFs with 5 μg/mL SE and T1DMs with 5 and 25 μg/mL SE. All wounds were closed by 24-hour, post-wounding. HKCs, T1DMs, and T2DMs with 25µg/mL SE exhibited a significant upregulation of cleaved vimentin compared to controls. Thrombospondin 1 was significantly upregulated in HCFs, HKCs, and T2DMs with 25 µg/mL SE. Lastly, HKCs, T1DMs, and T2DMs exhibited a significant downregulation of fibronectin with 25 μg/mL SE. Whether SEs can be utilized to clinical settings in restoring corneal defects is unknown. This is the first-ever study exploring the role of SEs in corneal wound healing. While the sample size was small, results are highly novel and provide a strong foundation for future studies.

## 1. Introduction

Exosomes are nanovesicles that originate from the inward budding of cell membranes that are released into the extracellular compartment [1,2,3]. While exosomes share common markers, including tetraspanins (i.e., CD9, CD63, and CD81), they also transport (i.e., cargo) tissue-specific biomolecules, such as DNA, mRNA, lipids, and proteins, allowing them to selectively influence the function of target cells [1,4,5,6]. In addition, exosomes carry anti-inflammatory markers, immunoglobulin A (IgA), and polymeric immunoglobulin receptor, highlighting their role in immunosuppression and wound healing processes [3,7,8]. The exosome cargo has been recently investigated for its diagnostic and therapeutic potential by several authors [9,10,11].

Exosomes are found throughout the body in blood [10], saliva [7,9], breast milk [12], amniotic fluid [13], and urine [13,14]. Using saliva to harvest exosomes offers a non-invasive, painless form of collection and does not require a large volume of sample for a suitable quantity of salivary exosomes (SE) [15,16]. SEs are preferable to whole saliva as they do not contain contaminating elements and carry lower amylase enzyme levels. Therefore, the interest in SEs as a diagnostic tool has recently increased, especially for systemic diseases [17,18,19,20,21]. To date, SE-related studies have been limited to systemic disease [22], and no reports exist in ocular disease(s).

Corneal wound healing is a complex cascade of events that involves epithelial, stromal, and endothelial cells (depending on the type/depth of injury) undergoing orchestrated proliferation, differentiation, and migration responses, ultimately leading to extracellular matrix (ECM) remodeling. The actual response to corneal injury varies among the three major corneal cell types. For instance, epithelial healing results in the differentiation and migration of limbal stem cells [23]; stromal healing is characterized by keratocyte transformation to fibroblasts and myofibroblasts [24]; and corneal endothelium primarily heals by cell migration with cell proliferation as a secondary response [25]. The release of exosomes from each corneal cell type appears to be involved in intracellular communication during homeostasis and wounding [26,27,28,29], thus impacting the wound healing process. For example, recent reports have shown mesenchymal stem cell (MSC)-derived exosomes promoting corneal epithelial wound repair [30] and reducing corneal fibrosis/inflammation [31].

Diabetes mellitus (DM) is associated with deficiency of the body to use blood glucose; however, complications can lead to delayed/inefficient wound healing compared to healthy populations. The diabetic cornea suffers from delayed wound healing [32,33], related to irregular fibrotic protein expression [34,35,36], inflammatory responses [37], and nerve defects. Additionally, despite published reports [22,38,39,40], the SE potential for therapeutic applications in the diabetic cornea remains unexplored.

Keratoconus (KC) is characterized by progressive corneal thinning, resulting in a cone-shaped corneal architecture and eventually leading to the loss of visual acuity due to corneal scarring [41,42]. KC is classically defined as a mainly degenerative non-inflammatory corneal disorder, with dysfunctions associated to oxidative stress and fibrotic markers [43,44]. However, several studies have provided evidence for inflammation as a driving factor for the pathogenesis of KC [45,46,47].

To our knowledge, this is the first report of the impact of SEs in the context of wound healing in healthy, DM, and KC corneal tissue. Future studies are warranted in order to delineate the potential therapeutic role of SEs related to corneal disease and trauma.

## 2. Results

### 2.1. Characterization of Salivary Exosomes

**Figure 1:**
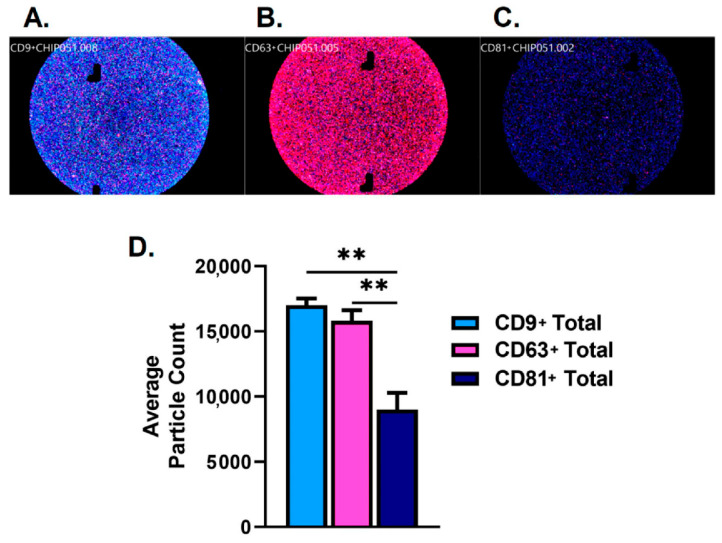
Fluorescent characterization of tetraspanins; CD9+, CD63+, and CD81+ from purified human SEs used for stimulation in this study. (**A**). Representative CD9+ fluorescence image of purified human SEs. (**B**). Representative CD63+ fluorescence image of purified human SE. (**C**). Representative CD81+ fluorescence image of purified human SEs. (**D**). Total particle characterization for CD9+, CD63+, and CD81+ of purified human SEs. ** *p* < 0.01.

Results of the purified human SEs demonstrated positive CD9(+), CD63(+), and CD81(+) expression. Figure 1A–C shows the representative images of each fluorescent channel used to detect tetraspanins CD9+, CD63+, and CD81+. CD81+ (9011) total particle counts were significantly lower when compared to CD9+ (17,015) and CD63+ (15,806) counts (Figure 1D).

**Figure 2:**
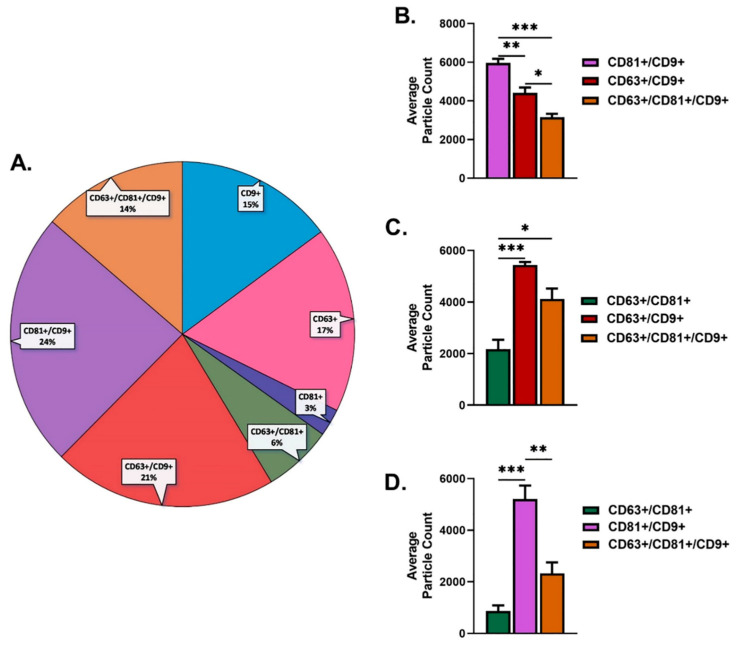
Colocalization characterization of tetraspanins; CD9+, CD63+, and CD81+ from purified human SE used for stimulation of cells in this study. (**A**). Percentage colocalization characterization for CD9+, CD63+, and CD81+. (**B**). CD9+ colocalization particle counts from purified human SEs. (**C**). CD63+ colocalization particle counts from purified human SEs. (**D**). CD81+ colocalization particle counts from purified human SEs. * *p* < 0.05, ** *p* < 0.01, and *** *p* < 0.001.

Proportions for overall CD9+ were 15%, CD63+ were 17%, and CD81+ were 3% (Figure 2A). Particle counts for CD9+ (15%) colocalization results showed that CD81+/CD9+ (24%) was significantly higher when compared to CD63+/CD9+ (21%) and CD63+/CD81+/CD9+ (14%) (Figure 2A,B). In addition, the particle count for CD63+/CD9+ (21%) was significantly higher when compared to CD63+/CD81+/CD9+ (14%) (Figure 2A,B). Colocalization particle count results for CD63+ (17%) showed that CD63+/CD81+ (6%) was significantly lower when compared to CD63+/CD9+ (21%) and CD63+/CD81+/CD9+ (14%) (Figure 2A,C). Finally, particle count for CD81+(3%) colocalization showed that CD81+/CD9+ (24%) was significantly higher when compared to CD63+/CD81+ (6%) and CD63+/CD81+/CD9+ (14%) (Figure 2A,D).

**Figure 3:**
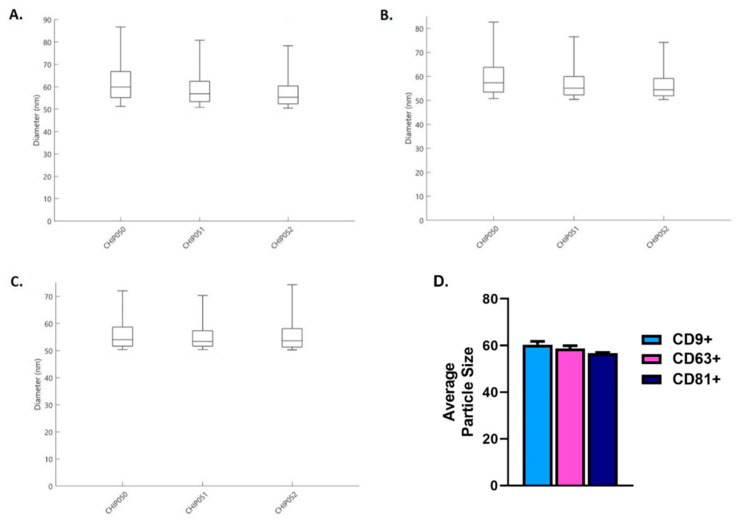
Particle size distribution of tetraspanins; CD9+, CD63+, and CD81+ from purified human SEs used for stimulation in this study. (**A**). CD9+ particle size of purified human SEs. (**B**). CD63+ particle size of purified human SEs. (**C**). CD81+ particle size of purified human SEs. (**D**). Average particle size for CD9+, CD63+, and CD81+. Note that labels CHIP050, CHIP051, and CHIP052 denote n = 3 for purified human SEs.

Exosomes’ sizes typically range from ~50 nm to 150 nm in diameter [9,48,49]. Our results showed that the particle size diameter range was ~50–90 nm for CD9+ (Figure 3A), ~50–80 nm for CD63+ (Figure 3B), and ~50–70 nm for CD81+ (Figure 3C). The combined particle size means for CD9+, CD63, and CD81 showed no significant difference (Figure 3D). The *x*-axis labels CHIP050, CHIP051, and CHIP052 denote n = 3 for the purified human SE.

### 2.2. Scratch Assay

**Figure 4:**
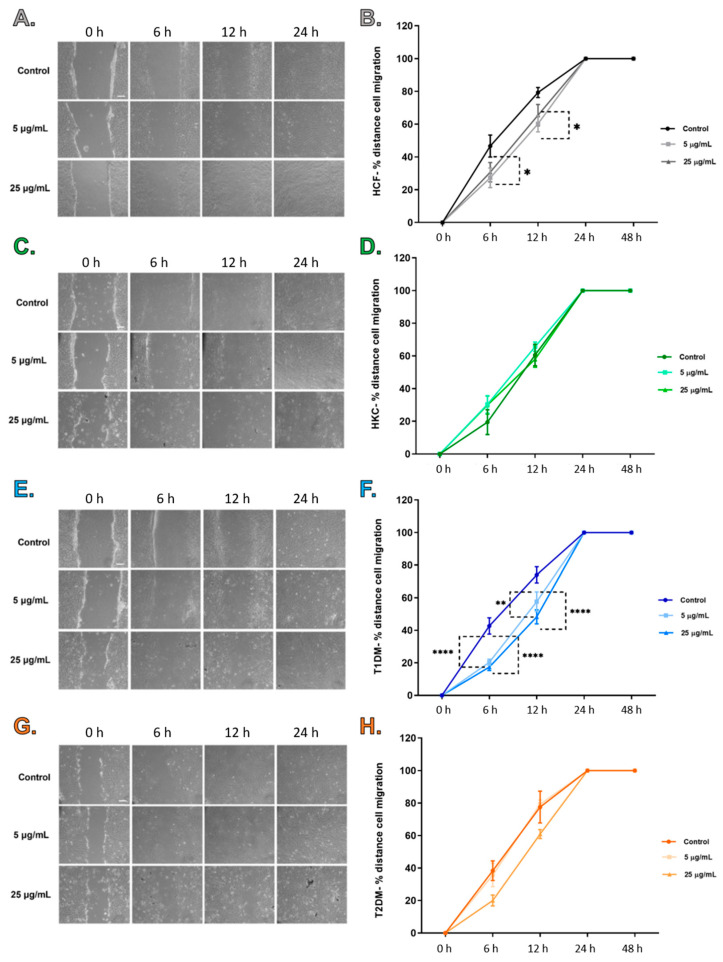
Scratch assay images at 0, 6, 12, and 24 h post-scratch assay are illustrated with a scale bar (white bar) of 200 μm. Percentage of total distant migration for each cell type taken at the reference points 0, 6, 12, 24, and 48 h after scratch assay was performed. SE stimulation with 5 and 25 μg/mL was performed at 0 h post-scratch assay, and no SE stimulation were considered control. (**A**). HCFs representative images. (**B**). Percentage of total distance migrated for HCFs (n = 3). (**C**). HKCs representative images. (**D**). Percentage of total distance migrated for HKCs (n = 3). (**E**). T1DMs representative images. (**F**). Percentage of total distance migrated for T1DMs (n = 3). (**G**). T2DMs representative images. (**H**). Percentage of total distance migrated for T2DMs (n = 3). * *p* < 0.05, ** *p* < 0.01, and **** *p* < 0.0001.

Scratch assays were performed to determine the effect of SEs on wound closure/migratory cell pattern of healthy (HCFs), KCs (HKCs), type I DM (T1DMs), and type II DM (T2DMs) human corneal fibroblasts cells (Figure 4). All four cell types achieved wound closure by 24 h post-wounding, including controls and SE-stimulated cultures (Figure 4). Images at 48 h (not included) showed complete wound closure similar to images shown at 24 h. Wound closure was measured and quantified at 0, 6, 12, 24, and 48 h. Control HCFs showed significantly higher cell migration when compared to HCFs stimulated with 5 μg/mL SEs at both 6 and 12 h (Figure 4B). There were no differences in cell migration for HKCs between the controls and the SE-stimulated groups (Figure 4D). At 6 and 12 h, control T1DMs showed significantly higher cell migration when compared to T1DMs stimulated with 5 and 25 µg/mL SEs (Figure 4F). Finally, no differences were observed for T2DMs between SE-stimulated groups and controls (Figure 4H). It can be concluded that SEs stimulation in control and T1DMs slow cell migration in the scratch assay at 6 and 12 h; however, the changes in migration were not observed at complete wound closure.

### 2.3. Protein Analysis

The protein expression of alpha-smooth muscle actin (α-SMA), thrombospondin-1 (TSP-1), fibronectin (EDA-Fn), vinculin, and vimentin was examined using Western blots for all cell types and conditions at 48 h. Beta actin (β-actin) expression was used to normalize data, and the mean ± SEM of four replicates (n = 3) was plotted. Expression of the native and cleaved form of vimentin was determined as previously reported [50,51]. Cultures with no SE stimulation served as controls.

**Figure 5:**
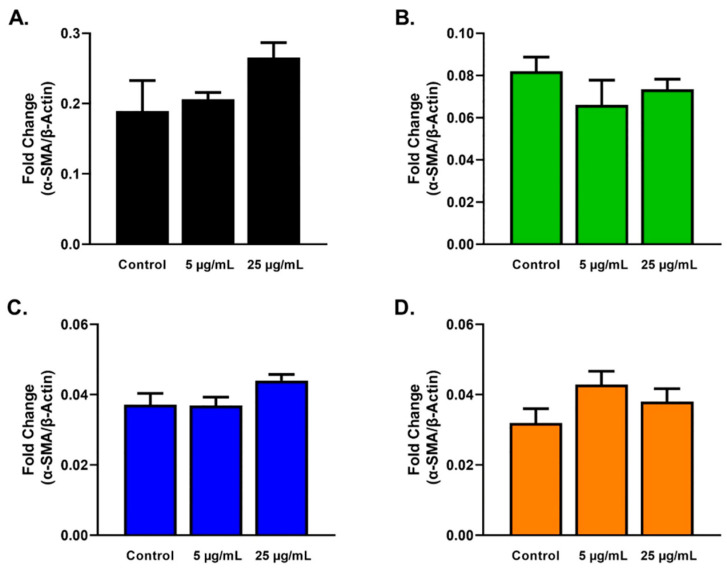
α-SMA expression in response to 5 and 25 µg/mL SE stimulation for 48 h after scratch assay performed in HCFs, HKCs, T1DMs, and T2DMs. (**A**). Expression of α-SMA in HCFs (n = 3). (**B**). Expression of α-SMA in HKCs (n = 3). (**C**). Expression of α-SMA in T1DMs (n = 3). (**D**). Expression of α-SMA in T2DMs (n = 3).

**Figure 6:**
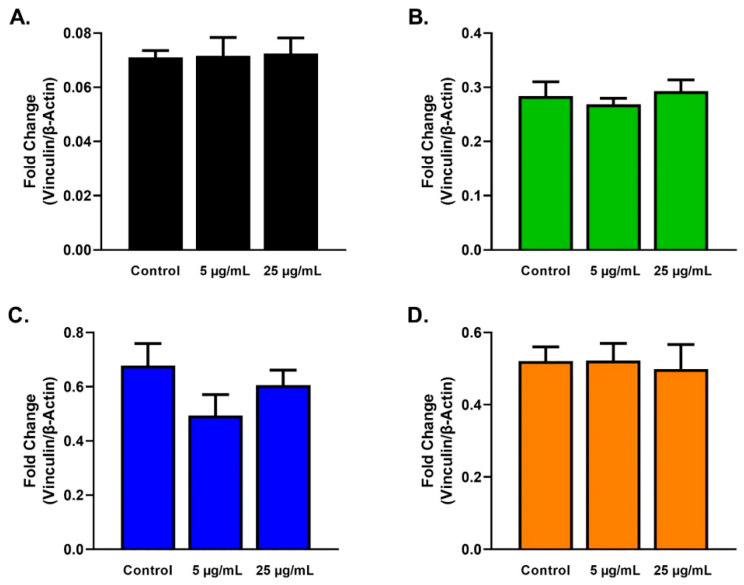
Vinculin expression in response to 5 and 25 µg/mL SE stimulation for 48 h after scratch assay performed in HCFs, HKCs, T1DMs, and T2DMs. (**A**). Expression of vinculin in HCFs (n = 3). (**B**). Expression of vinculin in HKCs (n = 3). (**C**). Expression of vinculin in T1DMs (n = 3). (**D**). Expression of vinculin in T2DMs (n = 3).

Expression of α-SMA (Figure 5) and vinculin (Figure 6) showed no significant differences for HCFs (Figure 5A and Figure 6A), HKCs (Figure 5B and Figure 6B), T1DMs (Figure 5C and Figure 6C), and T2DMs (Figure 5D and Figure 6D) cultures regardless of SE stimulation.

**Figure 7:**
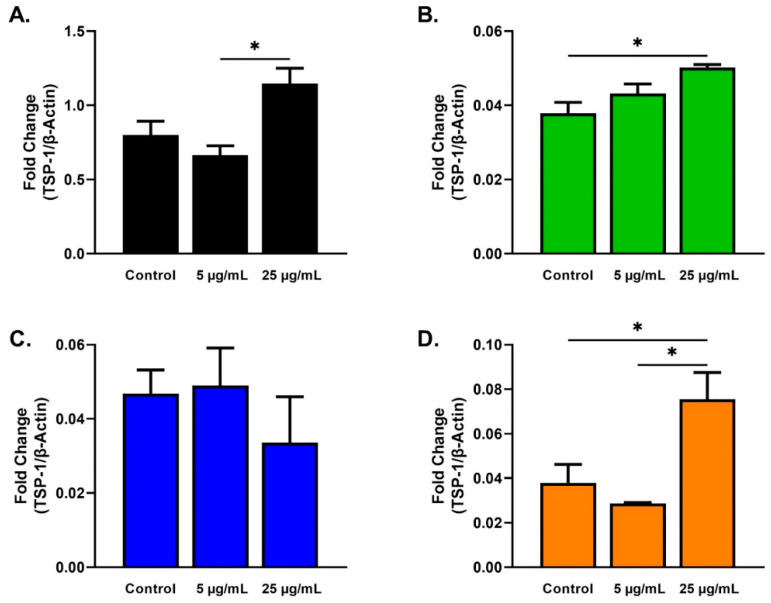
TSP-1 expression in response to 5 and 25 µg/mL SE stimulation for 48 h after scratch assay performed in HCFs, HKCs, T1DMs, and T2DMs. (**A**). Expression of TSP-1 in HCFs (n = 3). (**B**). Expression of TSP-1 in HKCs (n = 3). (**C**). Expression of TSP-1 in T1DMs (n = 3). (**D**). Expression of TSP-1 in T2DMs (n = 3). * *p* < 0.05.

The expression of TSP-1 was significantly higher for HCFs stimulated with 25 µg/mL SEs when compared to HCF stimulated with 5 µg/mL SEs (Figure 7A). For HKCs stimulated with 25 µg/mL, SEs showed significantly increased expression of TSP-1 when compared to HKCs controls (Figure 7B). Similar to HCFs, the expression of TSP-1 was significantly higher for T1DMs stimulated with 25 µg/mL SEs compared to T1DMs stimulated with 5 µg/mL SEs (Figure 7D).

**Figure 8:**
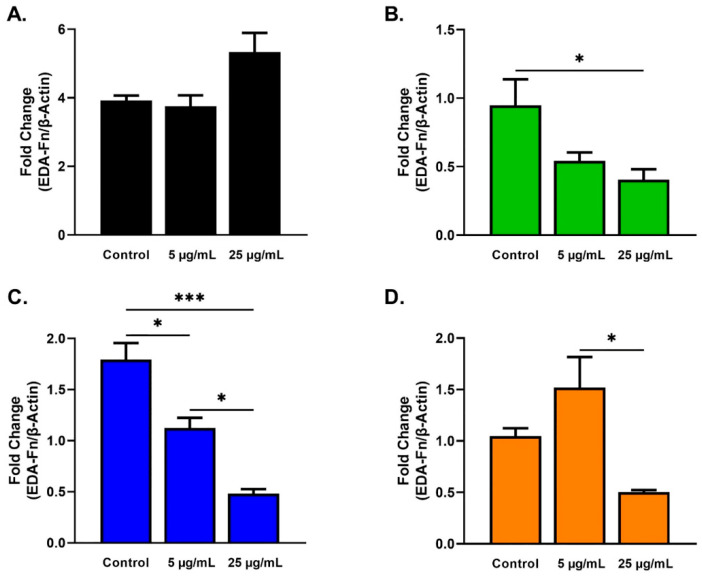
EDA-Fn expression in response to 5 and 25 µg/mL SE stimulation for 48 h after scratch assay performed in HCFs, HKCs, T1DMs, and T2DMs. (**A**). Expression of EDA-Fn in HCFs (n = 3). (**B**). Expression of EDA-Fn in HKCs (n = 3). (**C**). Expression of EDA-Fn in T1DMs (n = 3). (**D**). Expression of EDA-Fn in T2DMs (n = 3). * *p* < 0.05 and *** *p* < 0.001.

EDA-Fn expression differed significantly for HKCs, T1DMs, and T2DMs (Figure 8). EDA-Fn was significantly downregulated for HKCs stimulated with 25 µg/mL SEs when compared to HKCs controls (Figure 8B). Similarly, EDA-Fn expression was downregulated significantly for T1DMs stimulated with 5 and 25 µg/mL SEs when compared to T1DMs controls (Figure 8C). T1DMs stimulated with 25 µg/mL SEs showed a significant downregulated EDA-Fn expression when compared to T1DMs stimulated with 5 µg/mL SEs (Figure 8C). Lastly, EDA-Fn expression was also significantly downregulated for T2DMs stimulated with 25 µg/mL SEs when compared to T2DMs stimulated with 5 µg/mL SEs (Figure 8D).

**Figure 9:**
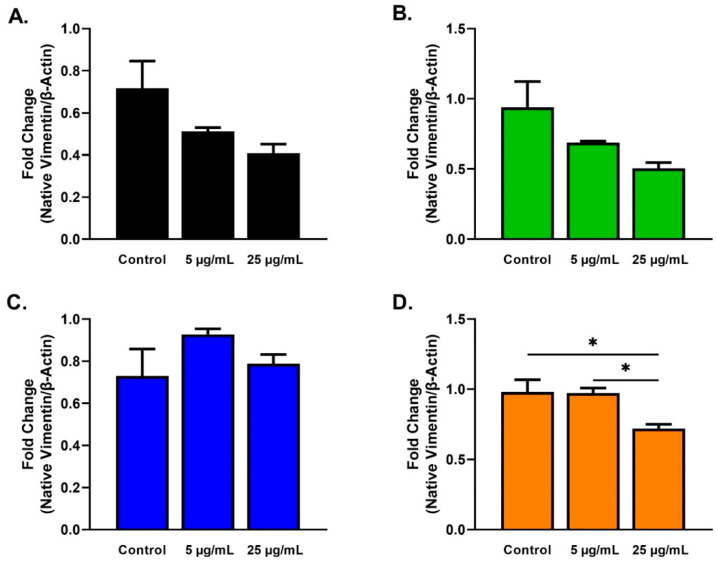
Native vimentin expression in response to 5 and 25 µg/mL SE stimulation for 48 h after scratch assay performed in HCFs, HKCs, T1DMs, and T2DMs. (**A**). Expression of native vimentin in HCFs (n = 3). (**B**). Expression of native vimentin in HKCs (n = 3). (**C**). Expression of native vimentin in T1DMs (n = 3). (**D**). Expression of native vimentin in T2DMs (n = 3). * *p* < 0.05.

Native vimentin expression was significantly decreased in T2DMs stimulated with 25 µg/mL SEs when compared to T2DM controls (Figure 9D). In addition, T2DMs stimulated with 25µg/mL SEs showed significant downregulation compared with T2DMs stimulated with 5 µg/mL SEs (Figure 9D).

**Figure 10:**
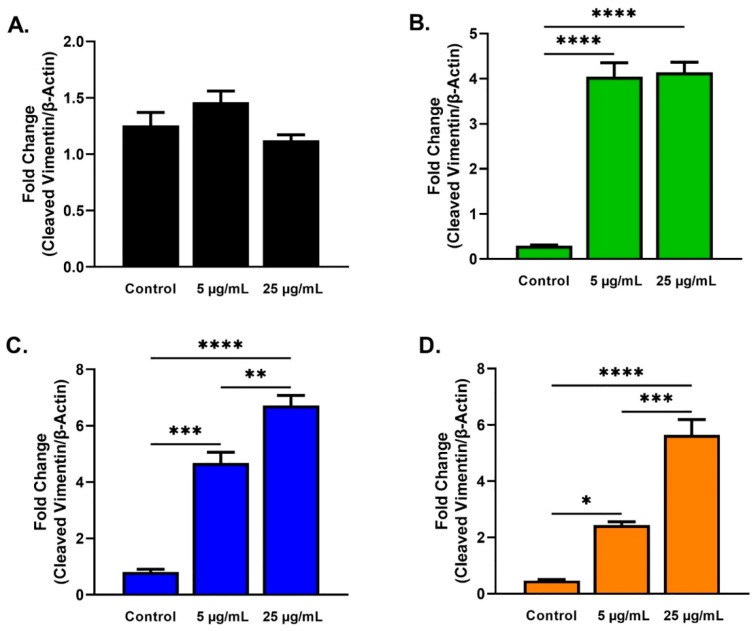
Cleaved vimentin expression in response to 5 and 25 µg/mL SE stimulation for 48 h after scratch assay performed in HCFs, HKCs, T1DMs, and T2DMs. (**A**). Expression of cleaved vimentin in HCFs (n = 3). (**B**). Expression of cleaved vimentin in HKCs (n = 3). (**C**). Expression of cleaved vimentin in T1DMs (n = 3). (**D**). Expression of cleaved vimentin in T2DMs (n = 3). * *p* < 0.05, ** *p* < 0.01, *** *p* < 0.001, and **** *p* < 0.0001.

The expression of cleaved vimentin showed significant differences for HKCs, T1DMs, and T2DMs (Figure 10). The cleaved vimentin expression in HKCs stimulated with 5 and 25 µg/mL SEs was significantly upregulated compared to HKCs controls (Figure 10B). Similarly, a significant upregulation of cleaved vimentin expression was observed for T1DMs and T2DMs stimulated with both concentrations of SEs when compared to their own controls (Figure 10C,D). Finally, a significant upregulation of cleaved vimentin expression was observed for T1DMs and T2DMs stimulated with 5 µg/mL SEs when compared to T1DM and T2DMs stimulated with 25 µg/mL SEs, respectively (Figure 10C,D).

## 3. Discussion

Exosomes are extracellular vesicles derived from body fluids and biological tissues/cells, transporting proteins, enzymes, DNA, RNAs, microRNAs, and lipids [4,5,6]. The capacity of exosomes to transport numerous genetic and proteomic information to recipient cells makes them essential players in intercellular communication. Thus, the interest in exosomes’ role as novel drug delivery vehicles has significantly increased over the last two decades, including reports in inflammation mechanisms [52,53], angiogenesis [54], cancer progression [55], and wound healing [56,57]. The role of exosomes in cell repair and wound healing has also been explored in vitro in skin [58,59,60], skeletal [61], cardiac [62], and corneal [29,30] settings.

Our current studies have focused on SEs due to their non-invasive collection methods and similar contents compared to other exosome forms [63]. However, it is currently unknown if SEs can promote tissue remodeling and repair during corneal wound healing. To our knowledge, this study is the first to explore SEs in the context of corneal wound healing mechanisms in healthy and diseased tissue types. Our study includes only one cornea per disease, each from a different donor. This small sample size is a limitation of our study. Both in keratoconus and diabetes, factors such as sex and duration of disease can have a significant effect. Undeniably, the corneal wound healing cascade is complex, with numerous factors contributing to the end result, including resident cells, growth factors, and the ECM microenvironment [23,24,64]. That having been said, a recent study found that sex does not affect corneal wound healing in rabbits after alkaline burn [65]. Nevertheless, future complementary studies should examine the impact of SEs on corneal stromal wound healing, taking into consideration disease stage, sex, and age. In any scenario, the ease with which SEs can be collected and isolated can facilitate their use as therapeutics in clinical practice. In addition, the use of SEs for clinical therapeutics can also be inexpensive and be a more natural approach for ocular wound healing.

Our study investigated the SE impact on HCFs, T1DMs, T2DMs, and HKCs. Both the diabetic and keratoconic cornea are known for their fibrotic phenotype, as well as their wound healing imbalances [43,66,67]. In a recent study, MSC-derived exosomes improve hepatic glucose and lipid metabolism in T2DM [38] as well as ameliorate autoimmune T1DM by reducing pancreatic islet destruction and increasing anti-inflammatory markers [39]. In addition to alleviating hyperglycemia, retinal pigmented epithelial cells derived exosomes in-vitro also slow the progression of chronic complications of diabetes, such as delayed cornea wound healing [40]. Interestingly, SEs [22] have been shown to alleviate diabetes-induced salivary gland dysfunction by reducing blood glucose levels, downregulating salivary amylase, salivary IgA, and serum nitric oxide in diabetic rats.

Additionally, a tear analysis of KC patients found decreased anti-inflammatory marker IL-10 and increased pro-inflammatory cytokines, including IL-6, tumor necrosis factor-α, and matrix metalloproteinase (MMP)-9 [45,46]. Inflammatory mediators, including chemokine ligand 5, MMP-13, IL-8, and IL-13 in tears have also been positively correlated with disease severity in keratoconic corneas [47]. In HKCs, we have previously observed low levels of keratocan, collagen I, and collagen V associated with myofibroblast differentiation [43,44]. HKCs also have elevated oxidative stress levels and increased fibrotic markers, such as collagen III, ultimately leading to fibrosis and scarring [43,44]. Additionally, our group also previously identified the significant downregulation of prolactin-induced protein in tears, a known multi-hormonal regulator, underlining the systemic role in KC [68]. Therefore, the selection of these diseases as our first SE investigation is scientifically justified.

Our study revealed that HKC and T2DMs stimulation with 25 µg/mL SEs promoted the upregulation of TSP-1 compared to their own controls, possibly resulting in ECM repair and corneal wound healing via the activation of transforming growth factor-beta 1 (TGF-β1; Figure 7B,D). In the cornea, TSP-1 has an essential role in wound healing by activating TGF-β1 [69,70,71], inducing healing by myofibroblast differentiation and ECM production [72,73]. It has been shown that following corneal wounding, TSP-1 is upregulated in epithelial, stromal, and endothelial cells at different stages of the wound healing cascade [73,74]. In addition, TSP-1-deficient mice exhibit corneal edema, opacity, and wound gaping after corneal incisions [74]. Interestingly, SE stimulation showed no significant difference in TSP-1 expression for T1DMs, highlighting the differences between the two types of DMs.

SE stimulation caused the significant downregulation of EDA-Fn in HKC, T1DM, and T2DM cells but not in HCFs (Figure 8B–D). EDA-Fn is another fibrotic marker which, like TSP-1, activates latent complexes of TGF-β. EDA-Fn mediates the formation of a provisional matrix in the wound area, which is critical for inducing epithelial migration, adhesion, and spreading during the first phase of wound healing [75,76,77]. In addition, the increased appearance of EDA-Fn in the injured corneal stroma in early phases and disappearance as wound healing progresses has been observed [75,78,79]. Our results suggest that the higher concentration of SE facilitates wound healing in the diseased through late stages of recovery when EDA-Fn expression is downregulated. EDA-Fn has been reported to promote corneal epithelial wound healing in diabetic rats by the administration of EDA-Fn eyedrops that can accelerate epithelial wound closure and delayed epithelial resurfacing [34]. It is plausible that by reducing EDA-Fn expression at targeted, advanced stages of corneal healing, SEs may protect against fibrosis and the worsening of diabetic lesions. Although limited information exists, it has been suggested that elevated EDA-Fn expression may also contribute to KC progression [80,81]. The distribution of EDA-Fn was detected in the anterior portion of KC corneas but not found in the anterior portion of healthy or scarred corneas [82]. Given that SEs led to a significant reduction of EDA-Fn expression in KCs, it might suggest a role in slowing down/controlling the fibrotic phenotype of advanced KC.

Our results showed increases in cleaved vimentin in SE-stimulated HKCs, T1DMs, and T2DMs, suggesting cleavage of the native vimentin into truncated forms promoting wound healing and cell migration (Figure 9 and Figure 10). As a cytoskeletal protein, vimentin is essential for tissue remodeling processes, including wound healing. Zhou et al. found that the expression of vimentin and tenascin, an ECM protein, was upregulated in KC corneas [80]. Still, it is unclear if the elevated vimentin detected was altered due to the KC progression or cleaved into insoluble products. On the other hand, the depletion of vimentin has also been found to dampen the fibrotic response, as measured by decreased α-SMA expression and regenerative healing in corneal alkali injury in mice [83]. Gan et al. illustrated the role of vimentin in the eye by performing a scratch wound assay on retinal pigment epithelium cells, showing that vimentin acts as a growth template for intermediate filaments to maintain cell polarity for directed migration [84]. Given vimentin’s extensive roles in wound healing and cell migration, SEs may trigger vimentin cleavage to promote wound healing in diseased cells due to the fibrotic dysfunction observed.

## 4. Conclusions

This study highlights the potential role of SEs related to corneal trauma/disease. Our study measured fibrotic marker expressions late in the wound healing process after wound closure was completed. Future studies should track the levels of fibrotic phenotypes throughout the cornea wound healing process to confirm if fibrotic markers expression varies in the early stages compared to later stages of healing. Further studies, including larger number of cornea samples, are needed to understand the impact of SE in other cell types, such as epithelial and endothelial, during corneal wound healing. In addition, SE stimulation using an organ culture and/or our established 3D model is needed in order to closer represent the natural corneal wound healing process. Finally, in vivo studies are necessary to understand better the impact SEs have on corneal wound healing. Regardless, our findings are the first to suggest a potential therapeutic role of SE in cornea wound healing and paves the way for future studies.

## 5. Materials and Methods

### 5.1. Ethical Consent

Tissue collection written consent was obtained following institutional and federal guidelines. The study was approved by the North Texas Regional Institutional Review Board (NTIRB; protocol #2020-030). A total of four corneas from four donors were used, including one from each condition: healthy (40 y/o, male), KC (64 y/o, female), T1DM (63 y/o, female), and T2DM (80 y/o, female). Healthy (National Donor Research Interchange (NDRI, Philadelphia, PA, USA), T1DM (Oklahoma Lions Eye Bank), Oklahoma City, OK, USA,, and T2DM (Oklahoma Lions Eye Bank, Oklahoma City, OK, USA) are cadaveric tissue donations. KC tissue donations are live penetrating keratoplasty transplants from Dean McGee Eye Institute (DMEI; University of Oklahoma Health Sciences Center, Oklahoma City, OK, USA).

### 5.2. Characterization of Salivary Exosomes

Commercially available SEs (EXOP-510A-1, System Biosciences, Palo Alto, CA, USA) were purchased and fully characterized with ExoView^®^ R100 platform (NanoView Bioscience, San Diego, CA, USA).

In brief, SE’s tetraspanin profiles were examined using an ExoView^®^ Tetraspanin kit (Nanoview Bioscience, San Diego, CA, USA), which contains microarray chips to capture and characterize CD9, CD63, and CD81. SEs were diluted 100-fold to optimize signal-to-background expression and split in triplicates. Then, 70 µL of the sample was added to three chips, placed on a plate holder, and incubated overnight at room temperature. The plates were then placed into the ExoView^®^ Chip Washer (Nanoview Bioscience, San Diago, CA, USA). After the initial wash, 250 µL of blocker/fluorescent solution (provided in ExoView^®^ Tetraspanin kits, Brighton, MA, USA) was added containing mouse anti-human: CD81 with Alexa488, CD63 with Alexa647, and CD9 with Alexa555, followed by a 2 h incubation. The chips were dried, scanned, and analyzed using the NanoView Scanner software for particle counts, colocalization counts, and size.

### 5.3. Corneal Fibroblast Cells Isolation

Primary human corneal fibroblast cells, HCFs, HKCs, T1DMs, and T2DMs were isolated from donor corneas. In brief, the cornea’s epithelial and endothelial layers were scraped off using a razor and rinsed with sterile phosphate buffer saline (PBS; ThermoFisher Scientific, Waltham, MA, USA). The cornea pieces were cut into ~2 × 2 mm and placed into a T-25 flask (ThermoFisher Scientific, Waltham, MA, USA) with Eagle’s minimum essential media, (American Type Culture Collection, Manassas, VA, USA), 10% fetal bovine serum (R&D Systems, Minneapolis, MN, USA), and 1% antibiotic-antimycotic (Life Technologies, Gran Island, NY, USA) and incubated at 37 °C in 5% CO_2_. The medium was changed every two days, and cells were passaged in new flasks, as needed. All described experiments were executed with cells passage 3 to 6.

### 5.4. Scratch Assay

All cell types were seeded in clear flat-bottom 6-well plates (ThermoFisher Scientific, Waltham, MA, USA) at a concentration of 5 × 10^5^ cells per well and grew confluent over 24 h incubation at 37 °C with 5% CO_2_.

Cultures (n = 3) were stimulated with two concentrations of purified SEs of 5 μg/mL (2.84 × 10^9^ particles in 2 mL media) or 25 μg/mL (1.42 × 10^10^ particles in 2 mL media). Cultures with no SEs (media only) served as controls (n = 3). The scratch assay was executed on confluent cultures by scratching each well’s center using a 10 μL pipette tip. The scratch was marked using a red sticker to serve as a reference point. To track the progression of cell migration and wound closure, images were taken at 0, 6, 12, 24, and 48 h post-wounding, using an ACCU-SCOPE microscope (EXI-310; ACCU-SCOPE Inc., Commack, NY, USA) equipped with an EXCELIS HDS camera (AU-600-HD) at 4× magnification. Images were analyzed using ImageJ (National Institutes of Health, Bethesda, MD, USA).

### 5.5. Protein Extraction and Quantification

After 48 h of SE stimulation, cells were lysed using 1× radioimmunoprecipitation buffer and protease inhibitor (RIPA-PI, Sigma-Aldrich, St. Louis, MO, USA). In brief, cultures were washed two times with PBS, followed by the addition of 100 µL of RIPA-PI. All cells were then scraped off the wells with cell scrapers (ThermoFisher Scientific, Waltham, MA, USA) and incubated for 30-min at 4 °C in an ice bucket. Finally, cell supernatants containing protein were collected following 15 min centrifugation at 12,000 RPM at 4 °C.

Protein was quantified using Pierce™ BCA Protein Assay Kit (23225; ThermoFisher Scientific, Waltham, MA, USA), and Pierce™ Bovine Serum Albumin standards (23208; ThermoFisher Scientific, Waltham, MA, USA), per manufacturer protocols. A BioTek EPOCH2 microplate reader (BioTek, Winooski, VT, USA) was used to measure the absorbances at 562 nm. Values were plotted using linear regression with the standards to calculate and normalize protein concentration.

### 5.6. Western Blot Assay

Western blots were run by SDS-PAGE at 225 V for 39 min in Novex 4–20% Tris-glycine Mini Wedge 12-well gels (Invitrogen, ThermoFisher Scientific, Waltham, MA, USA) and transferred to nitrocellulose membrane (NCM) using iBlot 2 Dry Blotting System (Invitrogen, ThermoFisher Scientific, Waltham, MA, USA). NCMs were then blocked using 1× Blocker^TM^ fluorescent buffer (ThermoFisher Scientific, Waltham, MA, USA) for 1 h. Following blocking, 1:500 dilution of a primary antibody including EDA-Fn (SAB4200784; Millipore Sigma, Burlington, MA, USA), TSP-1 (ab85762; Abcam, Cambridge, MA, USA), and α-SMA (ab5694; Abcam, Cambridge, MA, USA), β-actin (ab184092; Abcam, Cambridge, MA, USA), vinculin (ab207440; Abcam, Cambridge, MA, USA), or vimentin (ab92547; Abcam, Cambridge, MA, USA) was added overnight at 4 °C. The NCMs were washed three times with Tris-Buffer Saline with 1% tween (TBST) for 5 min, followed by a 1 h incubation with the secondary antibody (1:2000); donkey anti-rabbit AlexaFluor 568 (ab175470; Abcam, Cambridge, MA, USA), at room temperature. The NCMs were imaged with the iBright FL 15,000 imaging system (ThermoFisher Scientific, Waltham, MA, USA) and analyzed with the iBright analysis software (ThermoFisher Scientific, Waltham, MA, USA).

### 5.7. Statistical Analysis

GraphPad Prism was used for graphical representation and statistical analysis using one-way ANOVA (GraphPad Software, Inc., La Jolla, CA, USA). Data were normalized to β-actin expression, and the average (mean ± SEM) of four replicates (n = 3) was plotted. Differences were considered statistically significant when *p* < 0.05.

## Data Availability

The data presented in this study are available on request from the corresponding author.
